# Advances in antibiotic therapy in the critically ill

**DOI:** 10.1186/s13054-016-1544-6

**Published:** 2016-12-05

**Authors:** Rufu Jia, Linpei Jia

**Affiliations:** 1 Cangzhou Central Hospital, Cangzhou, Hebei Province China; 2 Department of Nephrology, The Second Hospital of Jilin University, 130041 Changchun, Jilin Province China

Vincent and colleagues discussed some of the key issues related to antibiotic management in the critically ill, including problems associated with timing, duration, and dosing of antibiotics [1]. In particular, the authors highlighted the importance of early diagnosis of infection and controversies about the use of combination or monotherapy and the duration of therapy [1]. We agree that decisions regarding the use of antibiotics should be made on an individual basis, e.g., according to the severity of the disease and local microbiological patterns [1]. However, some issues should be taken into account in determining an antibiotic therapy.

Vincent et al.’s review covered all infections in the critically ill but, with regard to the duration of antibiotic treatment, the authors referred to a guideline for the management of sepsis and septic shock only [2]. Dichotomy according to nosocomial or community-acquired infection seems necessary to make better decisions. Moreover, severe nosocomial infections like sepsis due to resistant Gram-negative bacteria and mild infections like community-acquired pneumonia caused by a susceptible microorganism may need different guidelines. Non-intensive care unit-acquired pneumonia has recently been proposed as a new clinical entity, as epidemiological data seem to be different between patients acquiring hospital-acquired pneumonia in the intensive care unit versus general wards [3].

Among others, the consciousness state of the critically ill seems important for the initiation or discontinuation of antibiotic treatment. Ventilator-associated pneumonia is the most frequent intensive care unit-related infection in patients requiring mechanical ventilation, and comatose patients present a high risk of early-onset ventilator-associated pneumonia [4]. For comatose patients who required mechanical ventilation, antibiotic prophylaxis at intubation lowers the incidence of ventilator-associated pneumonia [4]. In patients with ventilator-associated pneumonia due to non-fermenting Gram-negative bacilli, there appears to be a higher risk of recurrence following short-course therapy, i.e., a 7–8-day course [5].

Doctors in Western countries may say that it takes seven days to cure a common cold with medication but it will take a week to get better without it, but this advice does not apply to developing countries like China. Subsequent bacterial infections may prolong a common cold to as long as one month. The use of antibiotics varies substantially, even among developed countries. This discrepancy is particularly important for pediatric patients and the elderly with poor health. In conclusion, for antibiotic medication, general recommendations should be tempered by awareness of many local and specific factors in order to get the best effect from medicines.
