# Supplementary material for: Advances in antibiotic therapy in the critically ill
Source: Crit Care. 2016 Dec 5;20:393. doi: 10.1186/s13054-016-1544-6 (PMC5139014; doi:10.1186/s13054-016-1544-6)
Supplement: Related texts

# Reply: Authors’ response

**Authors:** Jean-Louis Vincent, Matteo Bassetti, Bruno François, Fabio S. Taccone

We thank Drs Jia and Jia for their interest in our recent review on antibiotic therapy [1]. Indeed, as mentioned briefly in our concluding paragraph, it is obvious that optimal antibiotic therapy—in terms of choice of antibiotic, dose, and duration of therapy—in critically ill patients will be influenced by multiple factors. Examples include the past history and age of the patient, the site and severity of infection, any previous prolonged exposure to antibiotics, likely causative organisms, concomitant therapies such as renal support, and local microbiological patterns, including bacterial resistance. Another important issue is the immune status of these patients [6]. Sepsis may significantly and rapidly alter cellular and humoral immune responses, such that hospital-acquired (and recurrent) infections may be secondary to a marked immunosuppressed state, which, together with insufficient tissue drug penetrations and high resistance levels, may further limit the definition of “adequate” antibiotic therapy and clinical response in this setting.

Drs Jia and Jia also mention that antibiotic “prophylaxis” may reduce the occurrence of ventilator-associated pneumonia in brain-injured patients [4]. Nevertheless, this strategy had no impact on survival or hospital stay and needs to be further evaluated in prospective studies before it can be considered as “standard of care”.

Regarding the last statement on the use of antibiotics in patients with a common cold in developing countries, we respectfully disagree. Recommendations to rationalize the use of antibiotics in patients with acute respiratory infections have the common objective of minimizing unnecessary antibiotics because “antibiotic pressure” is one of the factors triggering bacterial resistance. Antibiotics are widely misused in medicine, especially for viral infections. Should subsequent bacterial infection develop, appropriate targeted antibiotics should then be started.
